# Genetic diversity and ex situ conservation of *Loropetalum subcordatum*, an endangered species endemic to China

**DOI:** 10.1186/s12863-018-0599-6

**Published:** 2018-02-13

**Authors:** Bai-Jun Li, Jie-Yu Wang, Zhong-Jian Liu, Xue-Ying Zhuang, Jiu-Xiang Huang

**Affiliations:** 1 South China Limestone Plants Research Center, College of Forestry and Landscape Architecture, South China Agricultural University, Guangzhou, 510642 China; 2 Research Institute of Forestry, Chinese Academy of Forestry, Beijing, 100091 China; 3 Shenzhen Key Laboratory for Orchid Conservation and Utilization, The National Orchid Conservation Center of China and The Orchid Conservation and Research Center of Shenzhen, Shenzhen, 518114 China

**Keywords:** *Loropetalum subcordatum*, Genetic variation, Genetic structure, Ex situ population, SRAP, Plant conservation

## Abstract

**Background:**

*Loropetalum subcordatum* is an endangered species endemic to China that is characterized by narrow distribution, small population size, and delayed fertilization. However, the genetic diversity of the entire extant natural and ex situ populations has not been assessed to date. In this study, we evaluated the genetic diversity and structure of six natural populations and a single ex situ population (the only known ex situ population of *L. subcordatum*) using sequence-related amplified polymorphism data.

**Results:**

In total, 553 reliable DNA bands, of which 359 (63.28%) were polymorphic, were amplified by polymerase chain reaction with combinations of 15 primers. Low average gene diversity within populations and high genetic differentiation were detected in *L. subcordatum*. A Mantel test demonstrated that there was a positive correlation between genetic and geographic distances, indicating that significant genetic divergence was likely the result of geographic isolation among natural populations. Furthermore, based on genetic structure patterns, populations of *L. subcordatum* were divided into three clusters. Group 1 was composed of specimens from Libo, Guizhou Province (GZ) and Huanjiang, Guangxi Zhuang Autonomous Region (GX). Group 2 was composed of Mt. Wuguishan, Guangdong Province (GD). Group 3 was composed of three populations in Hong Kong Special Administrative Region. Additionally, clonal reproduction probably existed in GD population. According to the genetic information analysis and field survey, the ex situ population did not match its source population (GD) in terms of genetics, and its habitat was different from the original natural habitat. We observed that a few individual GD seeds were needed to improve ZS ex situ in the future.

**Conclusions:**

Compared to previous SRAP-based studies of endangered plants, *L. subcordatum* had extremely low average gene diversity within populations and high genetic differentiation among populations. At present, the unique ex situ population has not been successful due to non-representative samples being taken, a smaller population size, and man-made changes in habitat. Potential strategies are suggested to improve the conservation of this species.

**Electronic supplementary material:**

The online version of this article (10.1186/s12863-018-0599-6) contains supplementary material, which is available to authorized users.

## Background

Genetic diversity plays a key role in the survival and succession process of plant species, and is usually considered to be an estimate of extinction risk [1–3]. Species with high genetic diversity have more alleles, which means that they might possess higher adaptability to environmental changes [4]. Plant populations with low genetic diversity may suffer from reduced adaptive capacity [5–7], which can result in a gradual decrease in population size and eventual extinction of a population [8–10]. This situation usually presents itself in rare and endangered plant populations with narrow distributions, small population sizes, and geographic isolation [11–13]. Therefore, one of the main goals of conservation and restoration of rare and endangered plants is to preserve the genetic diversity of plant populations [14, 15]. Research on genetic diversity can provide important information for evaluating and guiding conservation efforts [16–18].

The major techniques to protect rare and endangered plants include in situ conservation, reintroduction, and ex situ conservation [19]. Ex situ conservation, which involves maintaining populations outside of their natural habitat, is not only the basis for reintroduction but also an important supplementary measure for in situ conservation [20, 21]. One of the main roles of ex situ conservation is to preserve plant genetic diversity [14, 22]. Understanding the genetic diversity of ex situ populations and their source can contribute to further evaluation and conservation of plant populations [23–25], such as sampling strategies, germplasm resource collection, and improvement of ex situ population [22].

**Fig. 1:**
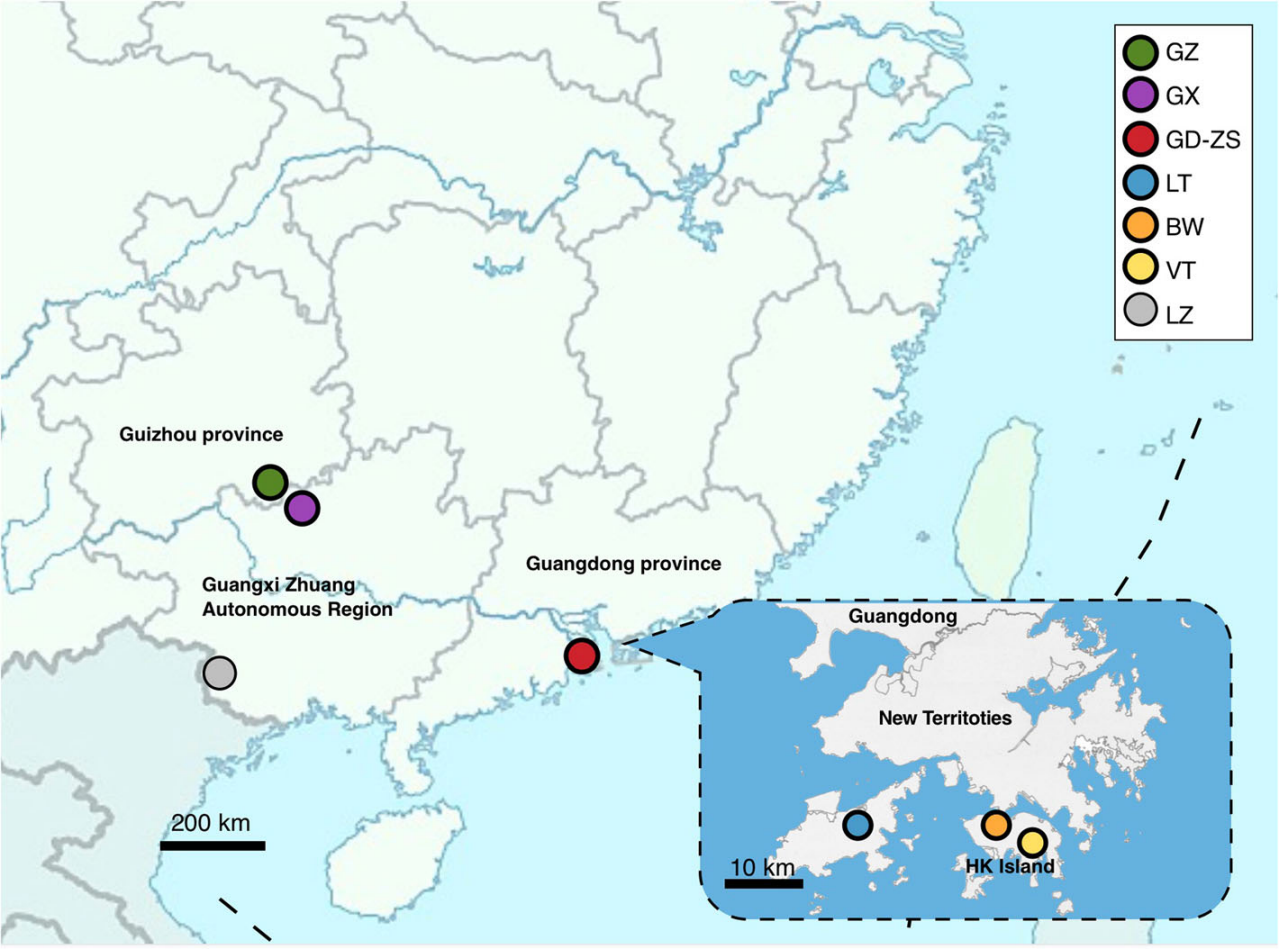
Distribution of *L. subcordatum*

*Loropetalum subcordatum* (Benth.) Oliv. (Hamamelidaceae) is a shrub or small tree that grows in evergreen broadleaved forests in southern and southwestern China. This species is self-pollinating and endemic to China. It has a low fruit set (4.79 ± 1.45%) in nature and a long-period of arrested development between flower senescence and ovary enlargement, which indicates that delayed fertilization occurs in this species [26]. According to records, *L. subcordatum* was distributed in Libo, Guizhou Province (GZ); Longzhou, Guangxi Zhuang Autonomous Region (LZ); Mt. Wuguishan, Guangdong Province (GD); and Bowen Road (BW) and Lantau Island (LT), Hong Kong Special Administrative Region (Fig. 1). However, the population in LZ was assumed to be extinct during field surveys several years ago [27]. According to our recent field investigations (Fig. 1), two new populations were found in Huanjiang, Guangxi Zhuang Autonomous Region (GX) and Violet Trail (VT), Hong Kong. GZ and GX had larger populations and inhabited high altitudes in the dry Karst mountains. Moreover, GD, BW, VT and LT were located in damp areas near streams at low altitude in the Granite Mountains or on islands. Because of its rarity and narrow distribution, *L. subcordatum* is classified as a National Protected Species (Class II) in China [28] and assessed as vulnerable based on the list of the International Union for Conservation of Nature [29]. Recently, the world’s only ex situ population was established with seeds from some individuals from GD at Zhongshan Arboretum, Guangdong Province (ZS) in order to protect the GD population. The ZS ex situ population inhabited a dry, latosolic, red soil hillside. In a previous study, an analysis based on amplified fragment length polymorphism (AFLP) was performed on the GZ, GD, BW and LT populations, which indicated that these four populations constituted four genetic clusters with respect to their genetic pool, and the two island populations (BW and LT) were likely to have come into existence via clonal reproduction [27]. And compared with previous AFLP-based studies of endangered plants, the results showed that these populations low genetic diversity (*Ht* = 0.22606, *Hw* = 0.1771) and high genetic divergence among populations (*Fst* = 0.72, *p* < 0.001), while no significant correlation was detected between genetic distance and geographic distance (*r*_*xy*_ = 0.854; *p* = 0.081) [27].

In this study, sequence related amplified polymorphism (SRAP) was conducted on populations of *L. subcordatum,* including those found in nature and the one conserved ex situ population, because it is a highly reliable genetic marker [30–33]. The aims of this study were to analyse the genetic diversity and structure of all known populations of *L. subcordatum* to establish a better understanding of genetic information about this species, to estimate the validity of ex situ populations and to propose potential measures for further conservation.

## Methods

### Sampling of L. Subcordatum

In this study, six natural populations (GZ, GX, GD, BW, LT, and VT) and one ex situ population (ZS) were analysed (Table 1; Fig. 1). According to the distribution of individuals in population, 10–15 individuals were randomly sampled from GZ, GX, BW, VT, and LT. In the field survey, adventitious roots germinating from stems immersed in stream water and growing in stone seams were observed in the GD and island populations, which indicated that vegetation propagation might exist. Only six samples were randomly taken from LT, since individuals were close to each other and we wished to avoid selecting potential clonal individuals. Additionally, the ZS ex situ was constructed in 2008 with seeds from some individuals of the GD population. Therefore, all the individuals of GD and ZS were collected in order to estimate whether the ZS ex situ could match its source population (GD) in terms of genetics. Juvenile leaves of individuals from seven populations were collected (Table 1) and stored in silica gel.

**Table 1:** Accession data for the six natural populations and one ex situ population of L*. subcordatum*

Pop abbr.	Locations	N	Population size	Longitude/ Latitude	Altitude (*m*)
GZ	Maolan Nature Reserve, Libo, Guizhou	15	More than 100	107°56″19′E 25°19”58’N	860
GX	Mulun Nature Reserve, Huanjiang, Guangxi	13	More than 100	107°55″35′E 25°8”31’N	800
GD	Mt. Wuguishan, Zhongshan, Guangdong	69	69	113°27″33′E 22°25”8’N	338
BW	Road Bowen, Hong Kong	10	Less than 30	114°10″44′E 22°16”1’N	140
VT	Violet Trail, Hong Kong	11	Less than 30	114°11″39′E 22°14”46’N	139
LT	Lantau Island, Hong Kong	6	Less than 30	113°56″49′E 22°16”03’N	205
ZS	Zhongshan arboretum, Zhongshan, Guangdong	27	27	113°22″32′E 22°29”30’N	77

*Pop abbr*., population *Abbreviation*; *N*, number of samples

### DNA extraction

Total genomic DNA was isolated from dried leaf tissues following the modified hexadecyl trimethyl ammonium bromide (CTAB) method [34]. The concentration and quality of the isolated DNA was examined and controlled using a Nucleic Acid and Protein Analyzer (Thermo Fisher Scientific Inc., The United States). The DNA extracts were diluted to 50 ng/μL for polymerase chain reaction (PCR) amplification.

### SRAP amplification

For PCR amplification, the procedure described by Li and Quiros [30] was used with some modifications. We selected 15 SRAP primer combinations from a total of 124 primers to produce clear and reproducible fragments [see Additional File 1], and these primers were used in the final SRAP analysis. PCR reactions were conducted in a total volume of 20 μL, consisting of 60 ng of DNA template, 3.5 mM of MgCl_2_, 0.25 mM of dNTPs, 2.5 U of Exprime *Taq*, 0.3 μM of each primer, and ddH_2_O. The PCR cycling program was set to 94 °C for 5 min, followed by 5 cycles of 35 °C for 1 min and 72 °C for 1 min. Then, the process continued for 30 cycles of 94 °C for 1 min, 35 °C for 1 min, and 72 °C for 1 min, followed by a final extension cycle of 72 °C for 10 min.

### Data analysis

All of the readable amplified fragments with the same gel mobility, which ranged from 100 bp to 2000 bp, with an occurrence frequency of greater than 5%, manually scored as present (1) or absent (0) for entry into a binary data matrix.

The binary data matrix was analyzed using POPGEN v.1.31 [35] to estimate genetic diversity parameters, including the percentage of polymorphic bands (*PPB*), Nei’s genetic diversity (*H*) [36], Shannon’s information index (*I*) [37], total gene diversity (*Ht*), the average gene diversity within populations (*Hw*), gene flow (Nm), and Nei’s genetic distance.

An unweighted pair-group method of the arithmetic (UPGMA) dendrogram tree was constructed based on Nei’s genetic distance using NTSYS 2.1 [38], and bootstrap values for nodal support were calculated using the UPGMA method in MEGA7 [39], in which the replication was set as 1000. We used STRUCTURE 2.1 [40] to analyse patterns in the genetic structure using a model based on genotype data and Bayesian methods to gather individuals to different clusters [41]. According to Gilbert et al. [42], the operating parameter of STRUCTURE 2.1 is the assumed populations (*K*) from 1 to 10 and 20 independent runs for each *K*, with a burn-in period of 1 × 10^5^ and 1 × 10^5^ Markov Chain Monte Carlo replicates after burn-in. Moreover, the admixture model and allele frequencies that correlated were chosen. *△K* was calculated based on the rate of change in LnP (D) between adjacent *K* [43]. The STRUCTURE analysis was conducted with the same operating parameter three times to see if the results were repeatable. Fixation Index-Statistics (*Fst*) as a measure of populations divergence was estimated using GenAlEx V.65 [44]. For natural populations and groups, a hierarchical molecular variance (AMOVA) was estimated and used for partitioning the genetic variance among groups, among individuals in different populations within groups, and among individuals within populations using GenAlEx V.65 [44]. In addition, based on the result of STRUCTURE 2.1, six natural populations were merged into three groups: (a) GZ and GX; (b) GD; (c) LT, VT, and BW. A Mantel test was used to detect the correlation between geographic and genetic distances using GenAlEx V.65 [44], calculating the geographic and genetic distances matrices after 999 permutations. We manually compared all of the individuals’ bands in each population to estimate the number of genotypes (*G*) and the ratio of genotypes to the number of sampled accessions (*G/N*). If the individuals in a population had the same band sequence that consisted of the presence (1) or absence (0) of SRAP fragments, they would have the same genotype.

## Results

### SRAP polymorphism and genetic diversity

**Fig. 2:**
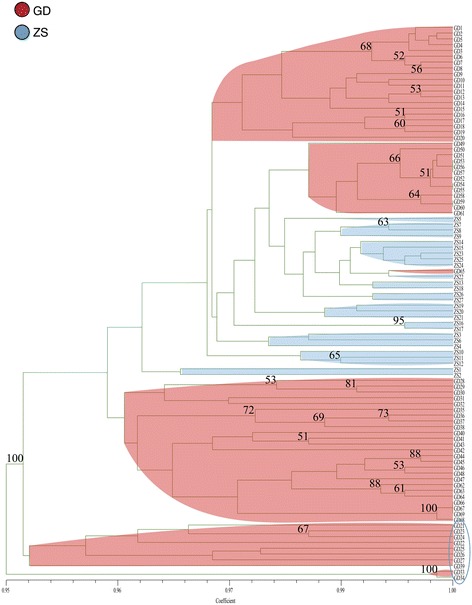
UPGMA dendrogram based on Nei’s genetic distance shows the genetic relationship between ZS ex situ and GD populations of *L. subcordatum*. The numbers near the nodes are bootstrap values that were higher than 50

A total of 553 reliable and reproducible DNA bands, ranging from 100 bp to 2000 bp, were amplified by PCR with the combinations of 15 primers [see Additional file 1], of which 359 (63.28%) were polymorphic with an average of 36.67 bands per primer pair. The coefficient of total gene diversity (*Ht*) was 0.2217, and the average gene diversity within populations (*Hw*) was 0.0469. Among the six natural populations, the percentage of polymorphic bands (*PPB*) ranged from 3.98% (in LT) to 21.7% (in GZ; Table 2). At the species level, Nei’s genetic diversity (*H*) was 0.1604, and Shannon’s information index (*I*) was 0.2581. At the population levels, the average values were as follows: *H* = 0.0469 (range of 0.0145–0.0904) and *I* = 0.0684 (range of 0.0217–0.1308) (Table 2). The number of genotypes was equal to the number of samples in GZ, GX, and three island populations (Table 2), but clonal reproduction possibly existed in GD (*G/N* = 0.913). The *PPB*, *H* and *I* of ZS were significantly less than those of GD (Table 2). Additionally, the result of the UPGMA dendrogram tree (Fig. 2) between GD and ZS showed that some individuals of GD were not contained in the ZS.

**Table 2:** A summary of the SRAP analysis of genetic variability of *L. subcordatum*

Population	*PPB* (%)	*H*	*I*	*G*	*G/N*
GZ	21.70	0.0904	0.1308	15	1
GX	17.00	0.0715	0.1031	13	1
GD	13.20	0.0447	0.0674	26	0.913
BW	7.78	0.0314	0.0455	9	1
VT	7.23	0.0290	0.0422	9	1
LT	3.98	0.0145	0.0217	4	1
Avg. in population level	11.82	0.0469	0.0684		
Species level	63.29	0.1604	0.2581		
ZS	7.59	0.0279	0.0415		
ZS/GD (%)	57.53	62.42	61.57		

*PPB*, the percentage of polymorphic bands (loci); *H*, Nei’s genetic diversity; *I*, Shannon’s information index; *G*, the number of genotype; and *G/N*. the ratio of genotypes to number of sampled accession

### Genetic differentiation

**Fig. 3:**
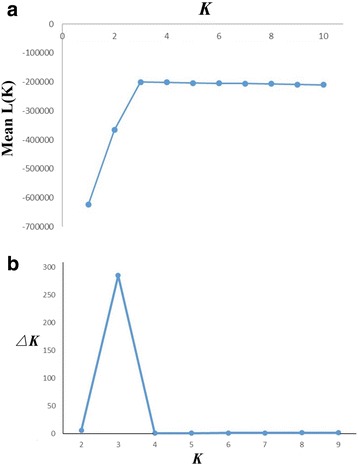
Estimated mean logarithmic likelihood of *K* values and relationship between the number of *L. subcordatum* populations (*K*) from 1 to 10 (**a**). The *△K* was calculated based on the rate of change in LnP(D) between adjacent *K* (**b**)

**Fig. 4:**
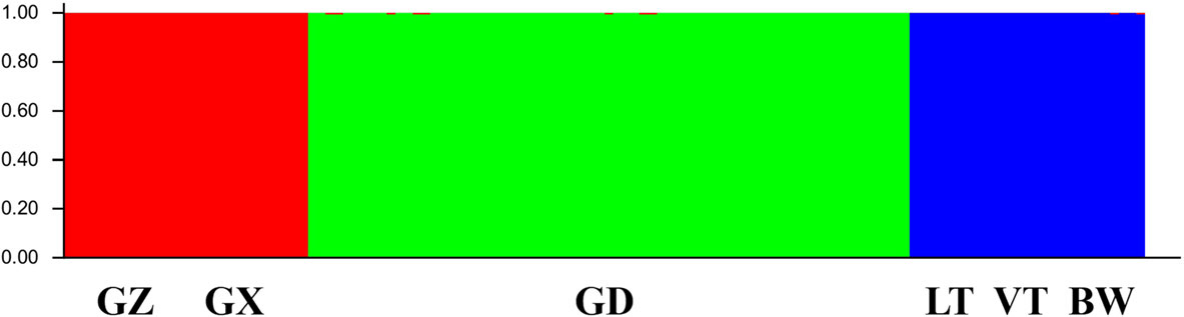
Proportional membership of 151 individuals from the one ex situ and six natural populations of *L. subcordatum* in the three clusters identified by STRUCTURE. Each individual is represented by a single *vertical bar*

**Fig. 5:**
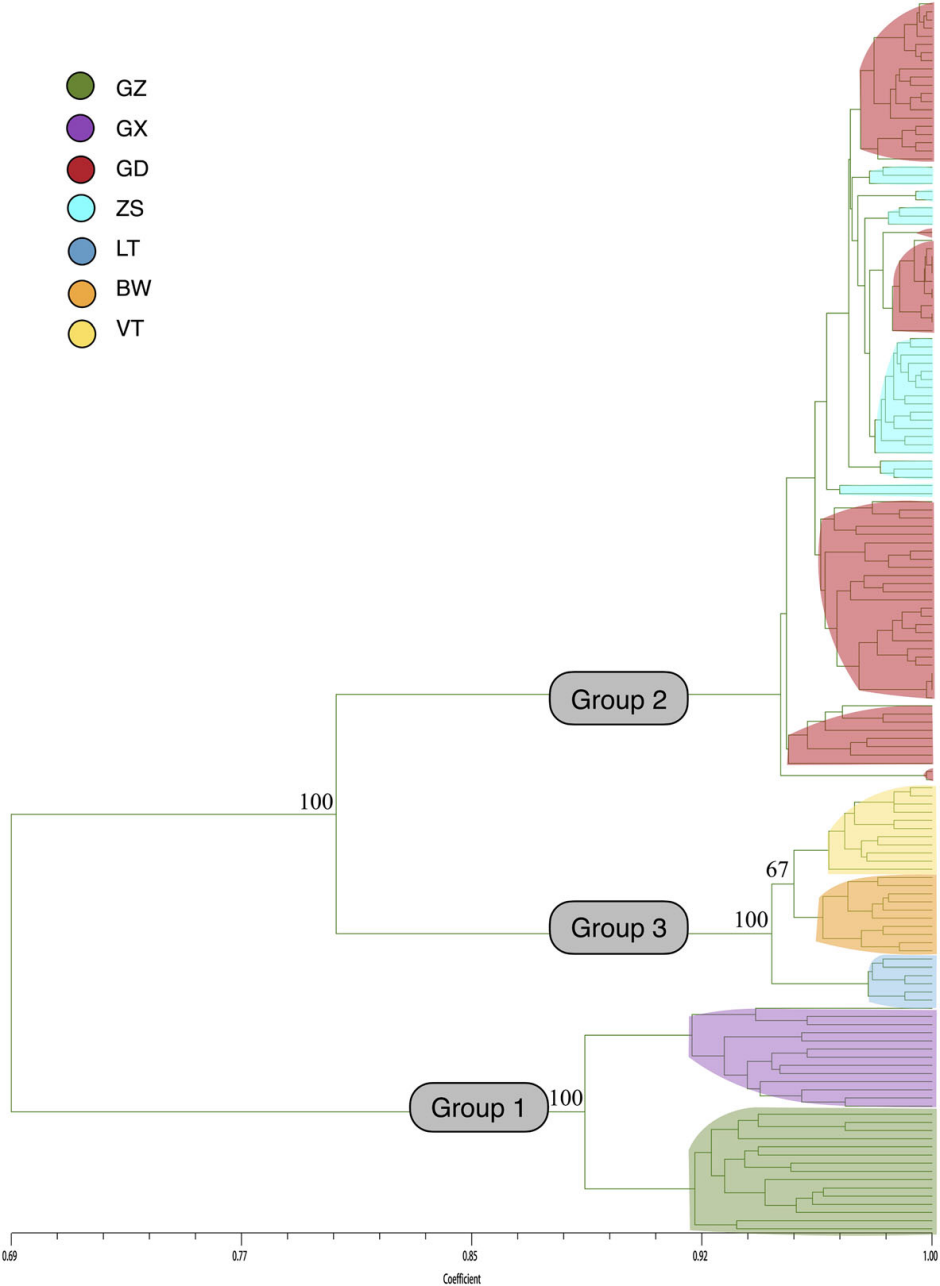
UPGMA dendrogram based on Nei’s genetic distance shows the genetic relationship among the six natural populations of *L. subcordatum*. The numbers in each node are bootstrap values and only the values of main clades are presented

**Fig. 6:**
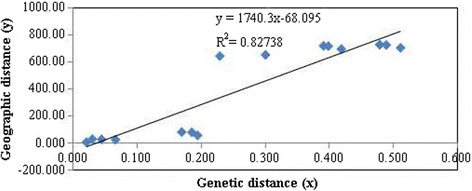
Relationship between genetic diversity (x) and geographic distance (y) among the six natural populations of *L. subcordatum*

Fixation Index-Statistics (*Fst* = 0.833) suggested that there was high genetic divergence among natural populations (Table 3). The pairwise estimates of *Fst* among natural populations were significantly different from zero (*p* < 0.001) and an additional file shows this information in more detail [see Additional file 2]. In addition, extremely low gene flow was detected (Nm = 0.1342). According to Evanno et al. [43], the optimal *K* was identified as 3 (Fig. 3a, b), i.e., an ideal genetic structural pattern would divide populations into three clusters (Fig. 4): (a) GZ and GX; (b) GD only; (c) LT, VT, and BW comprised the last cluster. This STRUCTURE result is repeatable. The hierarchical AMOVA showed that genetic variance was partitioned at 72% among groups, 13% among populations within groups, and 16% within populations (Table 3). Furthermore, the UPGMA dendrogram tree (Fig. 5) indicated the same genetic structure pattern as that identified by using STRUCTURE 2.1 software. Based on the SRAP data, a broad range of Nei’s genetic distance existed among the natural populations of *L. subcordatum*, varying from 0.0215 to 0.512 (Table 4). A Mantel test showed a significant positive correlation between genetic distance (x) and geographic distance (*r*_*xy*_ = 0.91, *p* = 0.01; Fig. 6) with a linear equation, y = 1740.3× - 68.095.

**Table 3:** The hierarchical analysis of molecular variance (AMOVA) examining differences among and within groups and natural populations of *L. subcordatum*

Source of variation	df	Sum of squares	Mean squares	Variance components	Percentage of variance components
Among groups	2	4342.642	2171.321	52.566	72
Among populations	3	330.437	110.146	9.420	13
within populations	118	1359.155	11.518	11.518	15
Total	123	6032.234		73.505	100

Six natural populations were merged into three groups: (a) GZ and GX; (b) GD; (c) LT, VT, and BW

**Table 4:** Nei’s unbiased measures of genetic identity and genetic distance among natural populations of *L. subcordatum* based on SRAP data

Pop ID	GD	VT	LT	GX	BW	GZ
GD	0.0000					
VT	0.1703	0.000				
LT	0.1952	0.0308	0.000			
GX	0.2297	0.3916	0.4193	0.000		
BW	0.1861	0.0215	0.0454	0.3991	0.000	
GZ	0.3011	0.4793	0.5120	0.0669	0.4895	0.000

Nei’s genetic distance (below diagonal)

## Discussion

### Genetic diversity of natural populations

Compared to previous SRAP-based studies of endangered plants, such as *Tetrastigma hemsleyanum* (*Ht* = 0.2407; *Hw* = 0.0731) [31], *Handeliodendron bodinieri* (*Ht* = 0.3127; *Hw* = 0.2402) [45] and *Cibotium barometz* (*Ht* = 0.2296; *Hw* = 0.1354) [46], the coefficient of total gene diversity (*Ht* = 0.2217) and the average gene diversity within populations (*Hw* = 0.0469) of *L. subcordatum* were very low. According to the field survey, *L. subcordatum* was vulnerable to fragmentation due to human disturbance and its low densities in forest habitat, which corresponds to previous knowledge about this tropical endemic tree [47]. Population fragmentation often results in a decrease in the number of individuals and an accompanying loss of genetic diversity [17]. Moreover, two features of *L. subcordatum*, self-pollination and low fruit set, were possible reasons for the constrained genetic diversity. The former feature usually reduces heterozygosity and decreases effective population sizes [48], while the latter feature obviously impedes demographic expansion. Thus, population fragmentation, autogamy, and fruit set are likely the reasons for the low genetic diversity of *L. subcordatum*. However, the genetic diversity measured by SRAP in this study was lower than that measured by AFLP in Gong et al. [27] (*Ht* = 0.22606, *Hw* = 0.1771, *PPB* = 11.36%~ 40%). A total of 47 samples and an average of 11 individuals of each population were used in Gong et al. [27]. However, more samples were collected in this study because two natural populations were added, and all of the individuals of GD were utilized to estimate ex situ and analyse genetic information. We collected samples which were evenly distributed in space for each natural population except the GD. We also conducted a test to ensure that when we applied the same collection method to choose 10–15 individuals of GD to compute using software, the result of this analysis would not be significantly different than those resulting from the inclusion of all individuals of GD. Therefore, it is possible that this discrepancy was caused by differences in the markers [49], as well as in the number of populations in the two studies, as the present study used more populations than were included in Gong et al. [27].

In addition, this study showed that the genetic diversity of the three continental populations (GZ, GX, and GD) was higher than that of the island populations (BW, VT, and LT; Table 2). Generally, compared with the continental populations, the island populations may have experienced long-term geographic isolation and genetic drift, which results in lower genetic diversity [50–52], and larger populations have more genetic variance [53, 54]. However, some species which have high dispersal ability did not demonstrate that their mainland populations had higher genetic diversity than island populations [55]. *L. subcordatum* possesses extremely low dispersal ability due to low fruit set and gravity-dispersed seed. Therefore, the differences between the continental and island populations may be a result of differences in population size, the island’s geographic isolation, and poor dispersal ability.

### Genetic structure of natural populations

In this study, the genetic differentiation among populations of *L. subcordatum* (*F*_*st*_ = 0.833) was higher than the findings of an earlier study (*F*_*st*_ = 0.72) by Gong et al. [27] and the average value for other autogamous plants (*G*_*st*_ = 0.523) [56]. Generally, high genetic divergence has been reported for many self-pollinated plants [16, 57], which can increase genetic drift and reduce gene flow [26, 58]. Unlike these species, seeds of *L. subcordatum* are gravity-dispersed, so they cannot spread over long distances, which decreases effective population sizes. Moreover, in addition to their breeding strategy, genetic drift, and gene flow, geographic distribution is also a major factor affecting genetic variance among populations [59].

*L. subcordatum* could be divided into three clusters (Figs. 4 and 5), which was supported by molecular variance among groups (Table 3) and that a higher genetic divergence occurred among groups. Additionally, the result of the Mantel test (*r*_*xy*_ = 0.91, *p* = 0.01; Fig. 6) was similar to previous studies [27] (*r*_*xy*_ = 0.854, *p* = 0.081) and showed that significant geographic isolation existed among populations. Therefore, we hypothesized that the geographic isolation of *L. subcordatum* populations resulted in extremely low gene flow (Nm = 0.1342) among natural populations, which made the species more susceptible to genetic drift and genetic differentiation [60]. Overall, the contributing factors to the high genetic differentiation of *L. subcordatum* were likely its reproductive strategy (autogamous), the geographic isolation of its populations, and the narrow distribution of its range.

### Evaluation of the genetic diversity of the ex situ population

ZS is the only extant ex situ population of *L. subcordatum* in China and is used as a source for protecting and improving the GD population. It has been suggested that 95% of a species’ alleles with an occurrence frequency of more than 5% should be included in ex situ populations for successful genetic conservation [61]. However, the genetic level of ZS (*PPB* = 7.59%; *H* = 0.0279; *I* = 0.0415) was much lower than that of GD (*PPB* = 13.2%; *H* = 0.0447; *I* = 0.0674), which suggests that the ex situ population does not adequately contain the genetic diversity of its source population. Additionally, the result of the UPGMA tree supported this analysis, and we found that ZS clearly derived from only part of the individuals of GD (Fig. 2). Meanwhile, the UPGMA tree can offer information about collecting extra seeds from some individuals of GD, which are shown in the blue circle (Fig. 2), to improve ex situ populations in the future. When conservationists established the ex situ, they randomly collected seeds from a portion of individuals of GD and did not yet know anything about the genetic information of the original population, which resulted in non-representative samples being taken. Moreover, ZS has only 27 individuals, which is too small: a limited founder number may cause a population bottleneck in ex situ populations [62, 63]. Additionally, the field survey showed that ZS ex situ was living on a dry, latosolic, red soil hillside, whereas GD was on a damp streamside on a granite mountain. These man-made changes in habitat are also more prevalent in the ex situ environment, increasing the genetic risk and weakening the conservation of *L. subcordatum* [64]. Therefore, ZS ex situ was not successful due to non-representative samples being taken, a smaller population size, and man-made changes in habitat.

### Clonal reproduction

The numbers of genotypes were equal to the number of samples in GZ, GX, and the three island populations. However, the genotype numbers were less than the sampling sizes found in GD (Table 2), which indicated that some identical genotypes existed, and these might be ascribed to vegetative propagation [27]. The GD population is located along the flood zone of a riparian area, suffers from the threat of habitat disturbance, and is vulnerable to damage: and its survival may depend on quickly establishing a new population. Clonal reproduction is a good strategy to allow for rapid demographic expansion [65]. Moreover, when sexual reproduction declines, such as a low fruit set or a lack of flowering under low-light conditions, and when habitat deteriorates, asexual propagation may arise to ensure population size and survival [66]. Therefore, the habitat alteration and decline in sexual reproduction were probably reasons to promote clonal reproduction in GD. Asexual propagation was not evident, and further studies are required to demonstrate the species’ mating system using molecular and physical methods. However, only two island populations (LT and BW) were likely to have clonality as shown in an earlier study [27]. The possible reason for these different results was that inconsistent samples were used in the two studies.

### Implications for conservation of *L. subcordatum*

As with other endangered species, another challenge to the survival of *L. subcordatum* populations is the influence of human activities. According to field surveys, BW is found around a city and VT distributes along both sides of a road used for tourism, while LT is crossed by a stream that contains a small dam further down its course. The habitat of GD is far from any city, but a small reservoir was built downstream, which prevents the spread of seeds via water flow and impedes population expansion. Moreover, based on our study, the unique ex situ population (ZS) was not successful and needs to be improved in the future due to its lower diversity, limited number of individuals, and unsuitable habitat. Efforts should be conducted to improve natural and ex situ populations in the future. All populations of *L. subcordatum* should be conserved in situ and ex situ, especially GD, BW, VT, and LT, by establishing protection zones to prevent interference introduced by human activity. Meanwhile, seed collection is a useful and effective method to build and maintain most ex situ populations [67, 68], and seeds from every individual of the natural populations of *L. subcordatum* should be collected as much as possible to store or use for future improvement of ex situ and in situ populations. Then, vegetative propagation can be used to effectively and rapidly expand ex situ population sizes. Additionally, a new habitat similar to the GD population’s living environment is required, and all of the existing individuals of the ZS ex situ should be transplanted into this new habitat. Finally, future studies should also focus on the mechanisms of the inherently low fruit set and delayed fertilization of *L. subcordatum*.

## Conclusions

The endangered species *L. subcordatum,* endemic to China, has low average gene diversity within populations and relatively high genetic differentiation. Based on an investigation of wild populations, we found that human activities impede the demographic expansion of *L. subcordatum* and fragment natural populations. The conservation of *L. subcordatum* is extremely urgent. The results of this study regarding the genetic information and UPGMA analysis between the ZS ex situ and its source population (GD) showed that this unique ex situ did not genetically represent GD and that the environment of the ex situ was different from its original habitat according to the field survey. Therefore, ZS ex situ conservation is not successful and needs to be improved in the future. Considering the previous discussion, more effective actions need to be taken to prevent further decline or extinction of *L. subcordatum*.

## Additional files

Additional file 1: Sequence of 15 SRAP selected primer combinations. (DOCX 14 kb)

Additional file 2: Results of pairwise population *Fst* analysis. (DOCX 14 kb)

## Abbreviations

AFLP: Amplified fragment length polymorphism
AMOVA: Analysis of molecular variance
BW: Bowen Road, Hong Kong Special Administrative Region, China
CTAB: Hexadecyl trimethyl ammonium Bromide
*Fst*: Fixation Index-Statistics
GD: Mt. Wuguishan, Guangdong Province, China
*Gst*: The coefficient of genetic differentiation
GX: Huangjiang, Guangxi Zhaung Autonomous Region, China
GZ: Libo, Guizhou Province, China
*H*: Nei’s genetic diversity
*Ht*: Total gene diversity
*Hw*: The average gene diversity within populations
*I*: Shannon’s information index
LnP (D): In likelihood of K
LT: Lantau Island, Hong Kong Special Administrative Region, China
LZ: Longzhou, Guangxi Zhuang Autonomous Region, China
N: Number of samples
Nm: gene flow
PCoA: Principal coordinate analysis
Pop abbr: Population abbreviation
*PPB*: The percentage of polymorphic band
SRAP: Sequence related amplified polymorphism
UPGMA: The unweighted pair-group method of arithmetic
VT: Violet Trail, Hong Kong Special Administrative Region, China
ZS: Zhongshan Arboretum, Guangdong Province, China
