# Supplementary material for: Genetic diversity and ex situ conservation of Loropetalum subcordatum, an endangered species endemic to China
Source: BMC Genet. 2018 Feb 13;19:12. doi: 10.1186/s12863-018-0599-6 (PMC5812050; doi:10.1186/s12863-018-0599-6)
Supplement: Supplementary file 1 — Sequence of 15 SRAP selected primer combinations. (DOCX 14 kb) [file 12863_2018_599_MOESM1_ESM.docx]

**Additional file 1.** Sequence of 15 SRAP selected primer combinations.

| No. | Code | Forward primer (5’-3’) | Reverse primer (5’-3’) |
| --- | --- | --- | --- |
| 1 | M7/E11 | 5’TGAGTCCAAACCGGTCC3’ | 5’GACTGCGTACGAATTGCA3’ |
| 2 | M7/E12 | 5’TGAGTCCAAACCGGTCC3’ | 5’GACTGCGTACGAATTCAT3’ |
| 3 | M12/E7 | 5’TGAGTCCAAACCGGAGG3’ | 5’GACTGCGTACGAATTGAG3’ |
| 4 | M12/E8 | 5’TGAGTCCAAACCGGAGG3’ | 5’GACTGCGTACGAATTGCC3’ |
| 5 | M12/E25 | 5’TGAGTCCAAACCGGAGG3’ | 5’GACTGCGTACGAATTCTG3’ |
| 6 | M13/E3 | 5’TGAGTCCAAACCGGAAA3’ | 5’GACTGCGTACGAATTGAC3’ |
| 7 | M13/E6 | 5’TGAGTCCAAACCGGAAA3’ | 5’GACTGCGTACGAATTGCA3’ |
| 8 | M15/E9 | 5’TGAGTCCAAACCGGAGA3’ | 5’GACTGCGTACGAATTTCA 3’ |
| 9 | M15/E11 | 5’TGAGTCCAAACCGGAGA3’ | 5’GACTGCGTACGAATTGCA3’ |
| 10 | M15/E17 | 5’TGAGTCCAAACCGGAGA3’ | 5’GACTGCGTACGAATTATG3’ |
| 11 | M15/E24 | 5’TGAGTCCAAACCGGAGA3’ | 5’GACTGCGTACGAATTCAG3’ |
| 12 | M16/E4 | 5’TGAGTCCAAACCGGATA 3’ | 5’GACTGCGTACGAATTTGA3’ |
| 13 | M16/E3 | 5’TGAGTCCAAACCGGATA 3’ | 5’GACTGCGTACGAATTGAC3’ |
| 14 | M16/E11 | 5’TGAGTCCAAACCGGATA 3’ | 5’GACTGCGTACGAATTGCA3’ |
| 15 | M16/E12 | 5’TGAGTCCAAACCGGATA 3’ | 5’GACTGCGTACGAATTCAT3’ |
