# Supplementary material for: Genetic diversity and ex situ conservation of Loropetalum subcordatum, an endangered species endemic to China
Source: BMC Genet. 2018 Feb 13;19:12. doi: 10.1186/s12863-018-0599-6 (PMC5812050; doi:10.1186/s12863-018-0599-6)
Supplement: Supplementary file 2 — Results of pairwise population Fst analysis. (DOCX 14 kb) [file 12863_2018_599_MOESM2_ESM.docx]

**Additional file 2.** Results of pairwise population Fst analysis.

| Pop ID | GD | VT | LT | BW | GX | GZ |
| --- | --- | --- | --- | --- | --- | --- |
| GD | 0.000 | 0.001 | 0.001 | 0.001 | 0.001 | 0.001 |
| VT | 0.816 | 0.000 | 0.001 | 0.001 | 0.001 | 0.001 |
| LT | 0.831 | 0.502 | 0.000 | 0.001 | 0.001 | 0.001 |
| BW | 0.825 | 0.387 | 0.576 | 0.000 | 0.001 | 0.001 |
| GX | 0.834 | 0.870 | 0.865 | 0.867 | 0.000 | 0.001 |
| GZ | 0.854 | 0.877 | 0.872 | 0.876 | 0.406 | 0.000 |

*Fst* values below diagonal. Probability, *p* values based on 999 permutations is shown above diagonal.
